# Association between Childhood Strabismus and Refractive Error in Chinese Preschool Children

**DOI:** 10.1371/journal.pone.0120720

**Published:** 2015-03-20

**Authors:** Hui Zhu, Jia-Jia Yu, Rong-Bin Yu, Hui Ding, Jing Bai, Ji Chen, Hu Liu

**Affiliations:** 1 First Clinical Medical College of Nanjing Medical University, Jiangsu Province, China; 2 Department of ophthalmology, Wuxi No.2 People's Hospital, Jiangsu Province, China; 3 Department of Epidemiology and Biostatistics, School of Public Health, Nanjing Medical University, Jiangsu Province, China; 4 Maternal and Child Healthcare Hospital of Yuhua District, Nanjing, China; 5 Department of Ophthalmology, Jiangsu Province Hospital, the First Affiliated Hospital with Nanjing Medical University, Jiangsu Province, China; Medical College of Soochow University, CHINA

## Abstract

**Purpose:**

To investigate the association between concomitant esotropia or concomitant exotropia and refractive error in preschool children

**Methods:**

A population-based sample of 5831 children aged 3 to 6 years was selected from all kindergartens in a representative county (Yuhuatai District, Nanjing, Jiangsu Province) of Nanjing, China. Clinical examinations including ocular alignment, ocular motility, visual acuity, optometry, stereopsis screening, slit lamp examination and fundus examination were performed by trained ophthalmologists and optometrists. Odd ratios (OR) and 95% confidence intervals (95% CI) were calculated to evaluate the association of refractive error with concomitant esotropia and concomitant exotropia.

**Results:**

In multivariate logistic regression analysis, concomitant esotropia was associated independently with spherical equivalent anisometropia (OR, 3.15 for 0.50 to <1.00 diopter (D) of anisometropia, and 7.41 for > = 1.00 D of anisometropia) and hyperopia. There was a severity-dependent association of hyperopia with the development of concomitant esotropia, with ORs increasing from 9.3 for 2.00 to <3.00 D of hyperopia, to 180.82 for > = 5.00 D of hyperopia. Concomitant exotropia was associated with astigmatism (OR, 3.56 for 0.50 to 1.00 D of astigmatism, and 1.9 for <0.00 D of astigmatism), myopia (OR, 40.54 for -1.00 to <0.00 D of myopia, and 18.93 for <-1.00 D of myopia), and hyperopia (OR, 67.78 for 1.00 to <2.00 D of hyperopia, 23.13 for 2.00 to <3.00 D of hyperopia, 25.57 for 3.00 to <4.00 D of hyperopia, and 8.36 for 4.00 to <5.00 D of hyperopia).

**Conclusions:**

This study highlights the close associations between refractive error and the prevalence of concomitant esotropia and concomitant exotropia, which should be considered when managing childhood refractive error.

## Introduction

Strabismus is a common childhood ocular disorder with population-based prevalence estimates ranging from 1.2% to 5% in Chinese children [1, 2]. The manifest misalignment of the eyes often results in deficient binocular depth perception and even amblyopia. Besides these functional effects, there are psychological distresses because of strabismus, such as depression and anxiety, impaired self-esteem and self-confidence, unsatisfied interpersonal relationships and social prejudice [3–7]. Surgical or optical therapy is necessary in many patients with strabismus.

The cause of strabismus has not yet been clearly understood and many factors may contribute. Children who were hyperopia in infancy have been found to be more likely to become strabismus [8]. Refractive accommodative esotropia has been identified to be the consequence of childhood hyperopia [9, 10]. However, how hyperopia influences other strabismus and how other types of ametropia influence strabismus are not known very well. Population-based research is significant in exploring the effect of refractive error on the development of various types of childhood strabismus. Confirming the relationship is very helpful to guide eye care providers to manage childhood refractive error and to influence public health policy-making. The present study aimed to analyze associations between refractive error and different types of strabismus in preschool children aged 3 to 6 years enrolled in the population-based Nanjing Pediatric Vision Project (NPVP).

## Methods

### Sample Selection

The NPVP was conducted from 2011 to 2012 and aimed to estimate the burden of common pediatric ocular disorders of preschool children aged 3–6 years in the Yuhuatai District, Nanjing, China. Nanjing City is the capital of Jiangsu Province, a traditional economic and cultural hub of eastern China and has a population of 8.1 million according to the China Sixth National Population Census (2010). Yuhuatai District is one of 11 municipal districts of Nanjing and has a relatively stable population structure (approximately 413 thousand residents) and a medium socioeconomic status in eastern China, which makes it representative of that area. The study adhered to the Declaration of Helsinki and was approved by the Institutional Review Board of Jiangsu Province Hospital. Written informed consent was obtained from parents or legal representatives of all participating children.

Every 150 to 250 children who studied in the 43 kindergartens in Yuhuatai District were grouped according to geographic location, which were defined as clusters. In total, 48 clusters were established, and every cluster had 200 children on average. All of the clusters were numbered according to their locations and were randomly selected using a random numbers table.

### Ocular Alignment and Movement

The eye examinations were performed by a team of two senior ophthalmologists, two junior ophthalmologists, two assistants and two optometrists. If glasses were worn, testing was performed with and without correction both. Ocular alignment was assessed using the Hirschberg light reflex test at a distance of 33 cm, the cover-uncover test and the alternate cover test with fixation targets at both 33 cm and 6 m [11]. Binocular and monocular ocular movements were examined at nine diagnostic positions of gaze with the head in a stationary position. If strabismus was suspected, a prism cover test was performed to detect the degree of eye misalignment.

### Distance Visual Acuity (VA)

All children had distance VA measured, with or without spectacles, using the Early Treatment Diabetic Retinopathy Study (ETDRS) visual acuity chart (Precision, Vision, LaSalle, IL, USA) at a distance of 4 m. For children with distance VA < 6/12 or a two or more lines difference between eyes, subjective refraction was performed to obtain best corrected VA.

### Optometry

All participants had the measurement of refractive error using an autorefractor (Suresight, Welch Allyn, USA) under non-cycloplegic conditions. The refraction status of children who were found abnormal in the examinations of ocular alignment, ocular movement and distance VA, was further evaluated under cycloplegic conditions if agreement was obtained from parents or legal representatives. One drop of topical 1.0% cyclopentolate (Cyclogyl, Alcon Pharmaceuticals) was administered to each eye twice at 5-minute intervals. Fifteen minutes later, a third drop of cyclopentolate was administered if the pupil size was < 6 mm or if the papillary light reflex was still present.

### Ocular Examination

Children with abnormalities found in the examinations of ocular alignment, ocular movement and distance VA needed to have further ocular examinations, including stereopsis screening using children random-dot stereograms (edited by Shaoming Yan, People’s Medical Publishing House, 2006, China), slit lamp examination, and fundus examination.

### Definition of Strabismus

Strabismus was determined if any tropia was present at distance or near, with or without spectacles and then classified according to the primary direction (esotropia, exotropia, vertical) of the tropia. Constant or intermittent heterotropia was defined as well.

### Statistical Analysis

Vector analysis was used to determine the J0 (power in the vertical or horizontal meridian) and J45 (power in the oblique meridian) vector components of astigmatism [12]. Potential risk factors were spherical equivalent (SE) refractive error of less hyperopia eye, astigmatism of less astigmatic eye, SE anisometropia, J0 anisometropia (interocular difference in J0), and J45 anisometropia (interocular difference in J45). The dioptric criteria for levels of magnitude are provided in Table 1. The less hyperopic eye was chosen for analysis because that accommodative convergence (a potential contributor to convergent strabismus) is likely to be caused by accommodation in the less hyperopic eye if anisometropia is present.

**Table 1:** Frequency Distributions of Refractive Error Risk Factors in 3 to 6 Years-Old Children with and without Strabismus in the Nanjing Pediatric Vision Project.

Risk factor	Esotropia	No Esotropia		Exotropia	No Exotropia	P Value^§^
	(N = 45), n(%)*	(N = 5786), n(%)*	P Value§	(N = 270), n(%)*	(N = 5561), n(%)*	
Age group						
3 y	3(0.5)	660(99.5)	0.137	25(3.8)	638(96.2)	0.02
4 y	17(0.7)	2466(99.3)		97(3.9)	2386(96.1)	
5 y	19(0.8)	2325(99.2)		133(5.7)	2211(94.3)	
6 y	6(1.8)	335(98.2)		15(4.4)	326(95.6)	
Sex						
Male	25(0.8)	3093(99.2)	0.881	147(4.7)	2971(95.3)	0.755
Female	20(0.7)	2693(99.3)		123(4.5)	2590(95.5)	
SE anisometropia(D)						
<0.50	23(0.4)	5573(99.6)	<0.001	195(3.5)	5401(96.5)	<0.001
0.50-<1.00	11(7.5)	135(92.5)		56(38.4)	90(61.6)	
> = 1.00	11(12.4)	78(87.6)		19(21.3)	70(78.7)	
J0 anisometropia(D)						
<0.25	36(0.6)	5612(99.4)	<0.001	213(3.8)	5435(96.2)	<0.001
0.25-<0.50	7(5.5)	120(94.5)		45(35.4)	82(64.6)	
> = 0.50	2(3.6)	54(96.4)		12(21.4)	44(78.6)	
J45 anisometropia(D)						
<0.25	40(0.7)	5673(99.3)	<0.001	238(4.2)	5475(95.8)	<0.001
0.25-<0.50	4(5.1)	74(94.9)		25(32.1)	53(67.9)	
> = 0.50	1(2.5)	39(97.5)		7(17.5)	33(82.5)	
SE refractive error(D)^†^						
<-1.00	1(4.3)	22(95.7)	<0.001	4(17.4)	19(92.6)	<0.001
-1.0-<0.00	0(0)	21(100)		7(33.3)	14(66.7)	
0.00-<1.00	16(0.3)	5334(99.7)		74(1.4)	5276(98.6)	
1.00-<2.00	6(2.4)	249(97.6)		138(54.1)	117(45.9)	
2.00-<3.00	8(6.7)	112(93.3)		35(29.2)	85(70.8)	
3.00-<4.00	2(7.1)	26(92.9)		9(32.1)	19(67.9)	
4.00-<5.00	2(12.5)	14(87.5)		2(12.5)	14(87.5)	
> = 5.00	10(55.6)	8(44.4)		1(5.6)	17(94.4)	
Astigmatism (D)^†^						
<0	14(4.4)	307(95.6)	<0.001	131(40.8)	190(59.2)	<0.001
0-<0.50	21(0.4)	5376(99.6)		114(2.1)	5283(97.9)	
0.5-<1.0	7(15.2)	39(84.8)		18(39.1)	28(60.9)	
> = 1.0	3(4.5)	64(95.5)		7(10.4)	60(89.6)	

D = diopters; J0 = power in the vertical or horizontal meridian; J45 = power in the oblique meridian; SE = spherical equivalent.

*Percentage of participants with stated outcome status.

§Chi square or Fisher exact test where applicable.

†Level of refractive error defined by the less hyperopic eye for SE refractive error, and the less astigmatic eye for astigmatic refractive error.

Binary logistic regression model was fitted to explore the associations of potential risk factors separately for concomitant esotropia and concomitant exotropia. Only age, gender and factors that were significant at the P<0.10 level were retained in further stepwise multiple logistic analyses. All analyses were performed using SPSS software (version 17.0, IBM, China) for Windows 7.0.

## Results

A total of 30 clusters including 5980 children were enrolled in this study and 5862 accepted eye examinations. Among the 5862 children, 31 were excluded from the data analysis for the following reasons: ages of 20 children were beyond the range of 3–6 years old, and 11 children had missing information. There were no significant differences in characteristics of children included in the data analysis compared with those excluded. Ultimately, 5831 children were analyzed, including 3118 boys (53.48%) and 2713 girls (46.52%), and the detection rate was 97.5%. The mean age of the children was 4.90±0.74 years.

337 children were found to have strabismus (prevalence, 5.8%, 95% CI 5.2%–6.4%) with no sex differences (5.9% [95%CI 5.07%-6.73%] in boys and 5.7% [95%CI 4.83%-6.57%] in girls). Among these, 6 had already undergone strabismus surgery and were orthophoria when examined. Of the 331 children having no history of surgery, 45 were concomitant esotropia, 270 were concomitant exotropia, 8 were microtropia, 7 were vertical strabismus and 1 were Duane retraction syndrome. The last three types of strabismus were excluded from analysis because of small numbers. Among children with concomitant esotropia, 80% were constant and 20% were intermittent. Among children with concomitant exotropia, 30% were constant and 70% were intermittent.

Agreement of cycloplegic optometry was obtained from parents or legal representatives of 370 children and refraction status of these children was measured under cycloplegic conditions. Among them, 41.6% had strabismus. The rest children only had the measurement of refractive error under non-cycloplegic conditions.

The univariate and multivariate analysis results for associations between refractive error and concomitant esotropia are provided in Table 2. After adjustment for other variables in the multivariate analysis, the following were identified as independent indicators of a greater risk for concomitant esotropia: SE anisometropia starting at the 0.50 to less than 1.00 D level (OR, 3.15–7.14 for different levels of SE anisometropia, relative to reference level of <0.50 D), and SE hyperopia starting at the 2.00 to less than 3.00 D level (OR, 9.28–180.82 for different levels of hyperopia, relative to reference level of 0.00 to <1.00 D).

**Table 2:** Univariate and Multivariate Analyses for Associations between Refractive Error and Childhood Concomitant Esotropia.

	Univariate				Multivariate*			
	OR	CI		p	OR	CI		p
Age group								
3 y	Ref				Ref			
4 y	1.52	0.44	5.19	0.507	1.37	0.36	5.17	0.638
5 y	1.80	0.53	6.09	0.346	2.12	0.57	7.88	0.264
6 y	3.94	0.98	15.85	0.054	2.70	0.54	13.53	0.227
Sex								
Male	Ref				Ref			
Female	0.92	0.51	1.66	0.779	0.85	0.43	1.64	0.62
SE anisometropia(D)								
<0.50	Ref				Ref			
0.50-<1.00	19.74	9.43	41.32	<0.001	**3.15**	**1.07**	**9.29**	**0.037**
> = 1.00	34.17	16.10	72.52	<0.001	**7.41**	**2.50**	**21.93**	**<0.001**
J0 anisometropia(D)								
<0.25	Ref				Ref			
0.25-<0.50	9.09	3.97	20.85	<0.001	1.14	0.40	3.27	0.802
> = 0.50	5.78	1.36	24.59	0.018	0.46	0.07	2.80	0.40
J45 anisometropia(D)								
<0.25	Ref				Ref			
0.25-<0.50	7.67	2.67	21.98	<0.001	1.83	0.53	6.36	0.344
> = 0.50	3.64	0.49	27.12	0.208	0.57	0.05	6.10	0.645
SE refractive error(D)								
0.00-<1.00	Ref				Ref			
-1.0-<0.00	0			0.999	0			0.998
<-1.00	15.15	1.93	119.28	0.01	3.70	0.31	43.70	0.299
1.00-<2.00	8.03	3.12	20.71	<0.001	3.15	0.82	12.10	0.095
2.00-<3.00	23.81	9.99	56.79	<0.001	**9.30**	**2.63**	**32.96**	**0.001**
3.00-<4.00	25.64	5.61	117.21	<0.001	**9.28**	**1.48**	**58.13**	**0.017**
4.00-<5.00	47.63	10.00	226.81	<0.001	**14.57**	**2.32**	**91.65**	**0.004**
> = 5.00	416.72	145.64	1192.33	<0.001	**180.82**	**36.37**	**898.89**	**<0.001**
Astigmatism (D)								
0.00-<0.50	Ref				Ref			
0.5-<1.00	45.95	18.47	114.33	<0.001	3.06	0.77	12.18	0.114
> = 1.00	12.00	3.49	41.24	<0.001	0.34	0.05	2.49	0.29
<0.00	11.67	5.88	23.18	<0.001	1.06	0.34	3.28	0.919

D = diopters; J0 = power in the vertical or horizontal meridian; J45 = power in the oblique meridian; SE = spherical equivalent.

Odds ratios in boldface are statistically significant.

*Adjusted for all factors retained in the mutivariate analysis.

Table 3 shows the univariate and multivariate analysis results for associations between refractive error and concomitant exotropia. In the multivariate analysis adjusted for all factors involved, SE hyperopia starting from the 1.00-<2.00 D level to the 4.00-<5.00 D level (OR, 8.36–67.78 for different levels of hyperopia, relative to reference level of 0.00 to <1.00 D), SE myopia starting at the -1.00 to less than 0.00 D level (OR, 18.93–40.54 for different levels of myopia, relative to reference level of 0.00 to <1.00 D), and astigmatism of 0.50 to less than 1.00 D and of less than 0.00 D (OR, 3.56 and 1.90 respectively, relative to reference level of 0.00 to <0.50 D) were identified as independent indicators of a greater risk for concomitant exotropia.

**Table 3:** Univariate and Multivariate Analyses for Associations between Refractive Error and Childhood Concomitant Exotropia.

	Univariate				Multivariate*			
	OR	CI		p	OR	CI		p
Age group								
3 y	Ref				Ref			
4 y	1.04	0.66	1.62	0.872	1.10	0.64	1.90	0.73
5 y	1.54	0.99	2.38	0.054	1.66	0.97	2.82	0.063
6 y	1.17	0.61	2.26	0.63	1.57	0.71	3.50	0.268
Sex								
Male	Ref				Ref			
Female	0.96	0.75	1.23	0.743	0.92	0.68	1.25	0.584
SE anisometropia(D)								
<0.50	Ref				Ref			
0.50-<1.00	17.23	11.99	24.77	<0.001	1.23	0.77	1.96	0.39
> = 1.00	7.52	4.44	12.73	<0.001	0.56	0.30	1.07	0.078
J0 anisometropia(D)								
<0.25	Ref				Ref			
0.25-<0.50	14.00	9.50	20.65	<0.001	1.06	0.66	1.73	0.80
> = 0.50	6.96	3.62	13.37	<0.001	0.49	0.23	1.07	0.073
J45 anisometropia(D)								
<0.25	Ref				Ref			
0.25-<0.50	10.85	6.63	17.76	<0.001	0.57	0.31	1.04	0.068
> = 0.50	4.88	2.14	11.14	<0.001	0.42	0.16	1.09	0.074
SE refractive error(D)								
0.00-<1.00	Ref				Ref			
-1.0-<0.00	84.09	60.06	117.75	<0.001	**40.54**	**13.16**	**124.86**	**<0.001**
<-1.00	29.36	18.61	46.30	<0.001	**18.93**	**5.25**	**68.22**	**<0.001**
1.00-<2.00	33.77	14.79	77.11	<0.001	**67.78**	**40.80**	**112.60**	**<0.001**
2.00-<3.00	10.19	2.27	45.61	0.002	**23.13**	**12.70**	**42.13**	**<0.001**
3.00-<4.00	4.19	0.55	31.93	0.166	**25.57**	**9.97**	**65.59**	**<0.001**
4.00-<5.00	35.65	14.00	90.88	<0.001	**8.36**	**1.71**	**40.97**	**0.009**
> = 5.00	15.01	4.99	45.20	<0.001	2.52	0.29	21.76	0.401
Astigmatism (D)								
0.00-<0.50	Ref				Ref			
0.50-<1.00	29.79	16.02	55.41	<0.001	**3.56**	**1.51**	**8.40**	**0.004**
> = 1.00	5.41	2.42	12.09	<0.001	0.42	0.16	1.08	0.071
<0.00	31.95	23.91	42.69	<0.001	**1.90**	**1.17**	**3.10**	**0.01**

D = diopters; J0 = power in the vertical or horizontal meridian; J45 = power in the oblique meridian; SE = spherical equivalent.

Odds ratios in boldface are statistically significant.

*Adjusted for all factors retained in the mutivariate analysis.

## Discussion

The present study used a large population-based cohort of children 3 to 6 years of age to identify the association between refractive error and childhood concomitant esotropia and concomitant exotropia. The major potentially risky refractive errors for concomitant esotropia were SE anisometropia of 0.5 D or more and hyperopia of 2.00 D or more. For concomitant exotropia, myopia, hyperopia of 1.00 to less than 5.00 D, hyperopic astigmatism of 0.50 to less than 1.00 D, and myopic astigmatism were independent risk factors. Several previous population-based studies also explored the relationship between refractive error and strabismus (Table 4) [1, 13, 14]. However, in SMS and STARS, a separate analysis of esotropia and exotropia was not reported. MEPEDS & BPEDS detected esotropia and exotropia separately and found that hyperopia and SE anisometropia are risk factors for esotropia and astigmatism increases the risk of developing exotropia, which is partially consistent with our data.

**Table 4:** Review of Population-based Studies of Association between Refractive error and Strabismus.

	Population	Association between refractive error and strabismus
SMS (Australia), Robaei (2006) [14]	1740 6-year-old school children examined between 2003–4; 26 (1.5%) with esotropia, 14 (0.8%) with exotropia	Myopia, hyperopia (> = 3 D), astigmatism (> = 1 D) and anisometropia (> = 1 D) were associated with strabismus in general (p <0.05). A separate analysis of esotropia and exotropia were not reported.
MEPEDS & BPEDS(USA) Cotter (2011) [13]	9970 children of African-American, Hispanic and non-Hispanic white children aged 6–72 months; 102 (1.0%) with esotropia and 102 (1.0%) with exotropia	For ET, hyperopia 2–<3 D, 3–<4 D, 4–<5 D and > = 5 D compared to 0-<1 D: OR 6.38, 23.06, 59.81 and 122.24, respectively (95% CI 2.56–15.93, 9.56–55.61, 23.61–151.52, and 49.86–299.7, respectively); SE anisometropia > = 1 D compared to <0.5 D: OR 2.03 (95% CI 1.01–3.73). For XT, astigmatism 1.5-<2.5 D and > = 2.5 D compared to <0.5 D: OR 2.49 and 5.88, respectively (95% CI 1.3–4.79 and 2.76–12.54, respectively); J0 anisometropia 0.25-<0.5 D and > = 0.5 D compared to <0.25 D: OR 2.01 and 2.63, respectively (95%CI 1.25–3.22 and 1.26–5.49, respectively).
STARS (singapore) Chia(2013)[1]	2992 Chinese children aged 6–72 months; 3 (0.1%) with esotropia and 20 (0.7%) with exotropia	Astigmatism > = 1 D compared to <1 D: OR 4.02 (95% CI 1.79–9.03); Anisometropia > = 1 D compared to <1 D: OR 7.16 (95% CI 2.08–24.67). A separate analysis of esotropia and exotropia were not reported.
This study (China)	5831 Chinese children aged 3–6 years; 45 (0.8%) with esotropia and 270 (4.6%) with exotropia	For ET, hyperopia 2–<3 D, 3–<4 D, 4–<5 D and > = 5 D compared to 0-<1 D: OR 9.3, 9.28, 14.57 and 180.82,respectively (95% CI 2.63–32.96, 1.48–58.13, 2.32–91.65, and 36.37–898.89, respectively); SE anisometropia 0.5-<1 D and > = 1 D compared to <0.5 D: OR 3.15 and 7.41, respectively (95% CI 1.07–9.29 and 2.5–21.93, respectively). For XT, myopia-1-<0 D and <-1 D compared to SE 0-<1 D: OR 40.54 and 18.93, respectively (95%CI 13.16–124.86 and 5.25–68.22, respectively); hyperopia 1-<2 D, 2–<3 D, 3–<4 D and 4–<5 D compared to 0-<1 D: OR 67.78, 23.13, 25.57 and 8.36, respectively (95% CI 40.8–112.6, 12.7–42.13, 9.97–65.59 and 1.71–40.97, respectively); astigmatism 0.5-<1.0 D and <0 D compared to 0–0.5 D: OR 3.56 and 1.9, respectively (95% CI 1.51–8.4 and 1.9–1.17, respectively).

MEPEDS, Multi-ethnic Pediatric Eye Disease Study; BPEDS, Baltimore Pediatric Eye Disease Study; SMS, Sydney Myopia Study; STARS, Strabismus, Amblyopia and Refractive Error in Singaporean Preschoolers Study

D = diopters; J0 = power in the vertical or horizontal meridian; SE = spherical equivalent; ET = esotropia; XT = exotropia

Esotropia has been found to occur more frequently in children with hyperopia than in those without and infants with moderate hyperopia have been reported to be more likely to develop esotropia than emmetropic controls [8, 15–21]. However, the risk of developing esotropia associated with different levels of hyperopia hasn’t been quantified well. Limited evidence-based researches have been conducted to explore this question. The present population-based data show that hyperopia starting at the 2.00 to less than 3.00 D level pose more than a 9-fold increase in concomitant esotropia risk and a marked rise in this risk is associated with nearly each diopter of increasing hyperopia (Table 2). The odds of having concomitant esotropia in children with hyperopia of 5.00 D or more are 180 times greater than in children with 0.00 to less than 1.00 D of hyperopia. These findings implied the importance of correction of childhood hyperopia starting at the level of 2.00 D. Eye care providers and parents might take advantage of the present data to make proper decisions about whether to monitor or correct childhood hyperopia, even though the benefits of prophylactic optical correction haven’t been determined.

In the case of exotropia, mild to moderate hyperopia showed a strong association with exotropia. Previous studies reported the association between hyperopia and the development of strabismus in general and did not analyze esotropia and exotropia separately [1, 14, 22]. There are few studies with which this risk association can be compared.

Anisometropia has been observed to reduce binocularity in people without strabismus [23, 24]. It makes the association between anisometropia and esotropia, which was shown from the present data, highly plausible. Previous studies also implied this kind of relationship even though they analyzed strabismus in general [14, 25, 26]. MEPEDS & BPEDS detected associations between anisometropia and esotropia and supported our findings strongly [13]. Anisometropia wasn’t found to be associated with exotropia in our study, while anisoastigmatism in the J0 component and exotropia were related in MEPEDS & BPEDS [13].

Myopia was shown to be related with the occurrence of exotropia in the present study. Data from a population-based observational study indicated that children with intermittent exotropia showed a significant trend toward myopia over time [27]. The reason of the association between intermittent exotropia and myopia hasn’t been clarified. One potential explanation is that intermittent exotropia might increase accommodative demand [28] and reduction of accommodation has been found to slow the progression of moderate myopia [29]. That is, intermittent exotropia may promote the development of myopia through increasing accommodation. Thus, we are unable to state that myopia is a risk factor for exotropia. Instead, it is likely that intermittent exotropia might be a risk factor for myopia. Further study is needed to clarify the link between myopia and exotropia.

Astigmatism was also related to exotropia in our analysis, which was reported in MEPEDS & BPEDS as well. However, MEPEDS & BPEDS detected association between the absolute astigmatism and strabismus, while we didn’t. Hyperopic astigmatism and myopic astigmatism are likely to have different effects on the development of strabismus. Therefore, it might be more reasonable to analyze them separately, which needs to be explored further in the future.

There are a number of limitations in our study. Most participants just underwent optometry under non-cycloplegic conditions, which might not reveal the real refraction status of these children, especially hyperopia. We just included age, gender and associated refractive errors into the multivariate analysis. It is possible that other unknown or unexplored factors also may contribute to strabismus. Because that confirmation of refractive error and alignment was available only for the time of clinical examination, older children’s refractive error may have been different from that at an earlier age when strabismus first occurred. Meanwhile, younger children who were at risk for strabismus might have yet to develop strabismus, which might lead to underestimate the strength of associations with refractive error. The principal strengths of the present study are the large cohort of children aged 3–6 years old from a representative area in China and the strict population-based design. Compared with clinic-based studies that might overrepresent severe disease, this research is more likely to explore real risk associations in the population. All participants received comprehensive eye examinations by qualified experienced ophthalmologists and optometrists who followed a standardized protocol of examination. Thus, it is less likely to misclassify strabismus and refractive error.

In conclusion, this population-based study of childhood strabismus found a strong link between refractive error and strabismus. It is helpful to guide eye care providers and parents in making informed decisions regarding management of early refractive error. However, further study is needed to clarify the association between refractive error and strabismus and to evaluate the benefits of prophylactic optical correction of early refractive error.
