# Correction: Association between Childhood Strabismus and Refractive Error in Chinese Preschool Children

**DOI:** 10.1371/journal.pone.0130914

**Published:** 2015-06-19

**Authors:** Hui Zhu, Jia-Jia Yu, Rong-Bin Yu, Hui Ding, Jing Bai, Ji Chen, Hu Liu

The references are listed out of order. Please see the complete, correct References list here.

1. Chia A, Lin XY, Dirani M, Gazzard G, Ramamurthy D, Quah BL, et al. Risk factors for strabismus and amblyopia in young singapore chinese children. Ophthalmic Epidemiology. 2013; 20(3): 138–147.

2. Fu J, Li SM, Liu LR, Li JL, Li SY, Zhu BD, et al. Prevalence of amblyopia and strabismus in a population of 7th-grade junior high school students in Central China: the Anyang Childhood Eye Study (ACES). Ophthalmic Epidemiology. 2014; 21(3): 197–203.

3. Lin S, Congdon N, Yam JC, Huang Y, Qiu K, Ma D, et al. Alcohol use and positive screening results for depression and anxiety are highly prevalent among chinese children with strabismus. Am J Ophthalmol. 2014; 157(4): 894–900.

4. Satterfield D, Keltner JL, Morrison TL. Psychosocial aspects of strabismus study. Arch Ophthalmol. 1993; 111(8): 1100–1105.

5. Menon V, Saha J, Tandon R, Mehta M, Khokhar S. Study of the psychosocial aspects of strabismus. J Pediatr Ophthalmol Strabismus. 2002; 39(4): 203–208.

6. Nelson BA, Gunton KB, Lasker JN, Nelson LB, Drohan LA. The psychosocial aspects of strabismus in teenagers and adults and the impact of surgical correction. J AAPOS. 2008; 12(1):72–76.

7. Xu J, Yu X, Huang Y, Chen J, Yu H, Wang Y, et al. The psychosocial effects of strabismus before and after surgical correction in Chinese adolescents and adults. J Pediatr Ophthalmol Strabismus. 2012; 49(3): 170–175.

8. Atkinson J, Braddick O, Robier B, Anker S, Ehrlich D, King J, et al. Two infant vision screening programmes: prediction and prevention of strabismus and amblyopia from photo- and videorefractive screening. Eye (Lond). 1996; 10(2): 189–198.

9. Raab EL. Etiologic factors in accommodative esodeviation. Trans Am Ophthalmol Soc. 1982; 80: 657–694.

10. Von Noorden GK, Campos EC. Binocular Vision and Ocular Motility: Theory and Management of Strabismus. 6th ed. St. Louis: Mosby; 2002.

11. Ojaimi E, Rose KA, Smith W, Morgan IG, Martin FJ, Mitchell P. Methods for a population-based study of myopia and other eye conditions in school children: the Sydney Myopia Study. Ophthalmic Epidemiol. 2005; 12(1): 59–69.

12. Miller J. Clinical applications of power vectors. Optom Vis Sci. 2008; 86(6): 599–602.

13. Cotter SA, Varma R, Tarczy-Hornoch K, McKean-Cowdin R, Lin J, Wen G, et al. Risk factors associated with childhood strabismus: the multi-ethnic pediatric eye disease and Baltimore pediatric eye disease studies. Ophthalmology. 2011; 118(11): 2251–2261.

14. Robaei D, Rose KA, Kifley A, Cosstick M, Ip JM, Mitchell P. Factors associated with childhood strabismus: findings from a population-based study. Ophthalmology. 2006; 113(7): 1146–1153.

15. Colburn JD, Morrison DG, Estes RL, Li C, Lu P, Donahue SP. Longitudinal follow-up of hypermetropic children identified during preschool vision screening. J AAPOS. 2010; 14(3): 211–215.

16. Robaei D, Rose KA, Ojaimi E, Kifley A, Martin FJ, Mitchell P. Causes and associations of amblyopia in a population-based sample of 6-year-old Australian children. Arch Ophthalmol. 2006; 124(6): 878–884.

17. Ip JM, Robaei D, Kifley A, Wang JJ, Rose KA, Mitchell P. Prevalence of hyperopia and associations with eye findings in 6- and 12-year-olds. Ophthalmology. 2008; 115(4): 678–685.

18. Anker S, Atkinson J, Braddick O, Ehrlich D, Hartley T, Nardini M, et al. Identification of infants with significant refractive error and strabismus in a population screening program using noncycloplegic videorefraction and orthoptic examination. Invest Ophthalmol Vis Sci. 2003; 44(2): 497–504.

19. Ingram RM, Arnold PE, Dally S, Lucas J. Results of a randomised trial of treating abnormal hypermetropia from the age of 6 months. Br J Ophthalmol. 1990; 74(3): 158–159.

20. Ingram RM, Walker C, Wilson JM, Arnold PE, Dally S. Prediction of amblyopia and squint by means of refraction at age 1 year. Br J Ophthalmol. 1986; 70(1): 12–15.

21. Atkinson J, Anker S, Bobier W, Braddick O, Durden K, Nardini M, et al. Normal emmetropization in infants with spectacle correction for hyperopia. Invest Ophthalmol Vis Sci. 2000; 41(12): 3726–3731.

22. Holmström G, Rydberg A, Larsson E. Prevalence and development of strabismus in 10-year-old premature children: a population-based study. J Pediatr Ophthalmol Strabismus. 2006; 43(6): 345–352.

23. Weakley DR. The association between nonstrabismic anisometropia, amblyopia, and subnormal binocularity. Ophthalmology. 2001; 108(1): 163–171.

24. Weakley DR. The association between anisometropia, amblyopia, and binocularity in the absence of strabismus. Trans Am Ophthalmol Soc. 1999; 97: 987–1021.

25. Huynh SC, Wang XY, Ip J, Robaei D, Kifley A, Rose KA, et al. Prevalence and associations of anisometropia and aniso-astigmatism in a population based sample of 6 year old children. Br J Ophthalmol. 2006; 90(5): 597–601.

26. O'Connor AR, Stephenson TJ, Johnson A, Tobin MJ, Ratib S, Fielder AR. Strabismus in children of birth weight less than 1701g. Arch Ophthalmol. 2002; 120(6): 767–773.

27. Ekdawi NS, Nusz KJ, Diehl NN, Mohney BG. The development of myopia among children with intermittent exotropia. Am J Ophthalmol. 2010; 149(3): 503–507.

28. Walsh LA, Laroche GR, Tremblay F. The use of binocular visual acuity in the assessment of intermittent exotropia. J AAPOS. 2000; 4(3): 154–157.

29. Chua WH, Balakrishnan V, Chan YH, Tong L, Ling Y, Quah BL, et al. Atropine for the treatment of childhood myopia. Ophthalmology. 2006;113(12);2285–2291.
